# Minimally Invasive Alveolar Ridge Preservation Utilizing an* In Situ* Hardening *β*-Tricalcium Phosphate Bone Substitute: A Multicenter Case Series

**DOI:** 10.1155/2016/5406736

**Published:** 2016-04-14

**Authors:** Minas D. Leventis, Peter Fairbairn, Ashish Kakar, Angelos D. Leventis, Vasileios Margaritis, Walter Lückerath, Robert A. Horowitz, Bappanadu H. Rao, Annette Lindner, Heiner Nagursky

**Affiliations:** ^1^Department of Oral and Maxillofacial Surgery, Dental School, University of Athens, 2 Thivon Street, Goudi, 115 27 Athens, Greece; ^2^Department of Periodontology and Implant Dentistry, School of Dentistry, University of Detroit Mercy, 2700 Martin Luther King Jr Boulevard, Detroit, MI 48208, USA; ^3^Dental College, Yenepoya University, University Road, Mangalore, Karnataka 575018, India; ^4^Medical School, University of Athens, 75 M. Assias Street, 115 27 Athens, Greece; ^5^PhD and DrPH Public Health Programs, College of Health Sciences, Walden University, 100 S Washington Avenue No. 900, Minneapolis, MN 55401, USA; ^6^Department of Prosthodontics, Preclinical Education and Materials Science, University of Bonn, Regina-Pacis-Weg 3, 53113 Bonn, Germany; ^7^Departments of Periodontics, Implant Dentistry, and Oral Surgery, New York University College of Dentistry, 345 E. 24th Street, New York, NY 10010, USA; ^8^Department for Oral and Maxillofacial Surgery, Cell Tissue Analysis (CTA), Medical Center, University of Freiburg, Hugstetter Street 55, 79106 Freiburg, Germany; ^9^Institute for Clinical Chemistry and Laboratory Medicine, Medical Center, University of Freiburg, Hugstetter Street 55, 79106 Freiburg, Germany

## Abstract

Ridge preservation measures, which include the filling of extraction sockets with bone substitutes, have been shown to reduce ridge resorption, while methods that do not require primary soft tissue closure minimize patient morbidity and decrease surgical time and cost. In a case series of 10 patients requiring single extraction,* in situ *hardening beta-tricalcium phosphate (*β*-TCP) granules coated with poly(lactic-co-glycolic acid) (PLGA) were utilized as a grafting material that does not necessitate primary wound closure. After 4 months, clinical observations revealed excellent soft tissue healing without loss of attached gingiva in all cases. At reentry for implant placement, bone core biopsies were obtained and primary implant stability was measured by final seating torque and resonance frequency analysis. Histological and histomorphometrical analysis revealed pronounced bone regeneration (24.4 ± 7.9% new bone) in parallel to the resorption of the grafting material (12.9 ± 7.7% graft material) while high levels of primary implant stability were recorded. Within the limits of this case series, the results suggest that *β*-TCP coated with polylactide can support new bone formation at postextraction sockets, while the properties of the material improve the handling and produce a stable and porous bone substitute scaffold* in situ*, facilitating the application of noninvasive surgical techniques.

## 1. Introduction

Tooth loss always leads to atrophic changes of the alveolar ridge and the key processes of postextraction bone modeling and remodeling have been well documented in both animal and human studies [1–3]. Human reentry studies showed horizontal bone loss of 29 to 63% and vertical bone loss of 11 to 22% during the first 6 months after tooth removal [4]. This three-dimensional resorption process at the postextraction sites may result in significantly narrowed ridges with reduced vertical height and lingual/palatal shifting of their long axes, rendering the subsequent correct placement of endosseous implants difficult or even impossible [5].

Recent evidence indicates that grafting the sockets at the time of tooth extraction, combined with “atraumatic” tooth removal and thorough debridement of the site, constitutes a predictable and reliable way to limit postextraction alveolar ridge resorption [6]. Such ridge preservation measures involve the use of a wide variety of bone substitutes, barrier membranes, and biologically active materials and several different surgical methods have been proposed [7]. However there is still no consensus regarding which material or technique is the most effective not only in limiting postextraction resorption but also at the same time in assisting the regeneration of high quality bone [6]. Bone substitutes, according to their origin, chemical composition, and biomechanical properties, show distinct biological behaviour regarding graft resorption (and/or dissolution) and new bone formation. This affects not only the* volume* but also the* quality* of the newly formed hard tissue, which is characterized by the bone architecture and the amount of vital bone, connective tissue, and residual graft it contains [7–12]. Theoretically, if bone* regeneration* is the aim of the surgeon's treatment, a fully resorbable material should be used so that the newly formed bone will be in all ways identical to the lost host bone. No residual graft should be present in the long term. Long-term incorporation of nonresorbable graft particles in the augmented bone will lead to* incomplete regeneration*, and in these cases* repair* is a more appropriate term [7, 13].

Alloplasts represent a group of synthetic, biocompatible bone substitutes. These biomaterials are free of any risk of transmitting infections or diseases by themselves, which might be an issue when utilizing xenografts or allografts. Moreover, their availability is unlimited in comparison to autogenous bone. One of the most promising groups of alloplastic bone substitutes is porous calcium phosphate ceramics. Apart from being osteoconductive, there is strong experimental evidence that calcium phosphates also have osteoinductive properties [14, 15]. Among ceramics *β*-TCP is commonly used [16–20]. Coating such alloplastic graft granules with PLGA can enhance the handling properties of the material and produce an* in situ *hardening, stable, and at the same time porous and osteoconductive bone graft substitute that serves as a scaffold for bone regeneration. From a clinical perspective, a self-stabilizing graft may greatly reduce the need for membranes to retain loose graft material in the defect, resulting in shortened, less expensive, and simplified surgical approaches [21, 22].

The bone quality of healed grafted sockets may influence the primary stability of implants placed into these regenerated sites [8]. Primary stability is believed to play an essential role in successful osseointegration and may lead to adequate secondary stability, thus contributing to the long-term implant success [23, 24]. Measurement of the final seating torque and resonance frequency analysis (RFA), noted as implant stability quotient (ISQ), are the most common noninvasive clinical test methods to assess the primary stability of the placed implant [23, 25].

It would be of great benefit to investigate if resorbable* in situ* hardening alloplastic grafting materials could be used in a minimally invasive way for postextraction ridge preservation and subsequent implant placement in a successful and predictable way. The purpose of the present report was therefore to investigate the hard and soft tissue healing of human postextraction sites grafted with an* in situ* hardening *β*-TCP alloplastic bone substitute without membrane coverage or soft tissue primary closure and to assess the initial stability of implants inserted into those sites at a 4-month time point.

## 2. Materials and Methods

### 2.1. Patients

**Figure 1:**
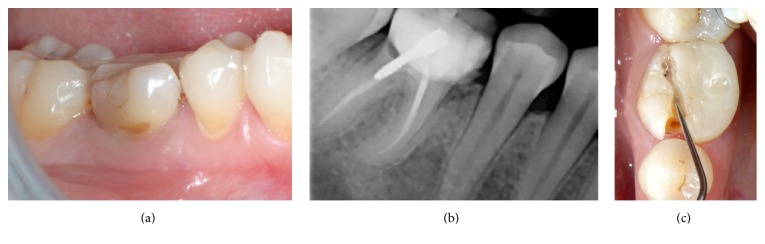
Nonrestorable mandibular first molar. (a) Initial clinical situation; (b) periapical X-ray; (c) occlusal view showing the mesiodistal fracture.

Between October 2012 and March 2014, ten patients who required extraction of a maxillary or mandibular tooth and subsequent single-tooth implant placement have been documented within the routine treatment by the surgeons. All patients were systemically healthy at the time of consultation. Smokers were included. The reasons for extraction included endodontic treatment failures and advanced caries lesions and fractures (Figure 1). Standard exclusion criteria for bone grafting procedures were applied including allergy, systemic chronic disease, alcoholism and drug abuse, pregnancy, or nursing mothers.

The case collection was performed in 3 different implantology clinics. Three different surgeons were involved. In each patient, the same surgeon performed all surgical procedures. All patients were monitored and examined clinically every 2 weeks during the healing period according to the standard routine treatment. In one case, it was possible to evaluate and document the soft tissue healing cascade of the site on a more regular basis.

In all patients periapical X-rays were taken before extraction, immediately after extraction and grafting, and after 4 months.

### 2.2. Surgical Procedures

**Figure 2:**
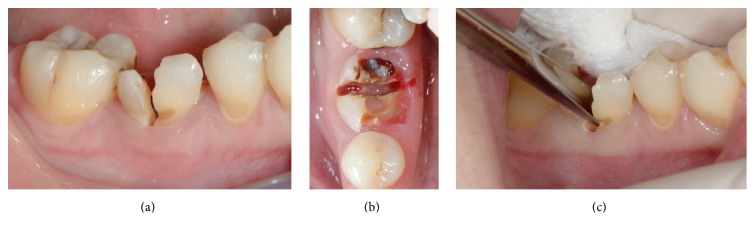
“Atraumatic” extraction carried out without flap elevation. Sectioning of the roots, buccal (a) and occlusal (b) view; (c) mobilization and removal of each root separately using a thin elevator.

**Figure 3:**
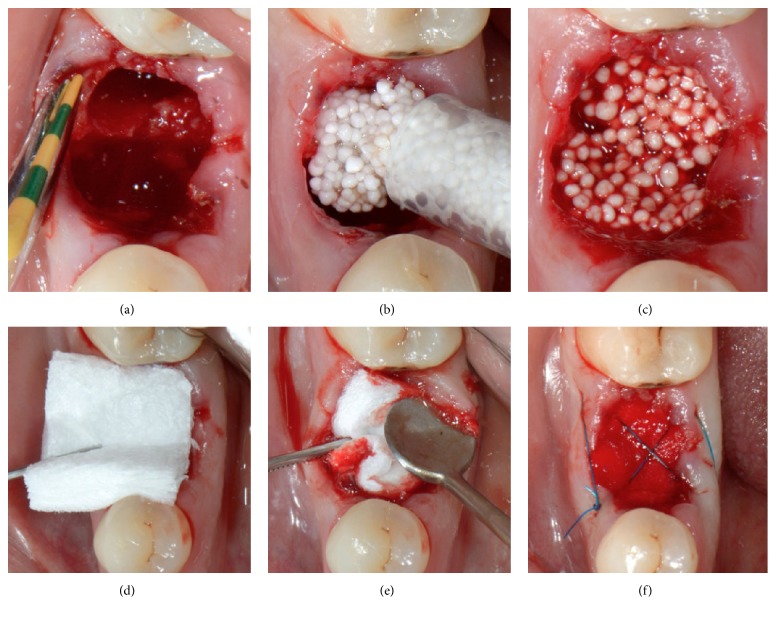
Socket grafting. (a) The integrity of the buccal plate was evaluated with a probe; socket filled with the* in situ *hardening bone substitute (b, c) and covered with a haemostatic dressing material (d, e); cross-mattress sutured (f) without primary closure.

**Figure 4:**
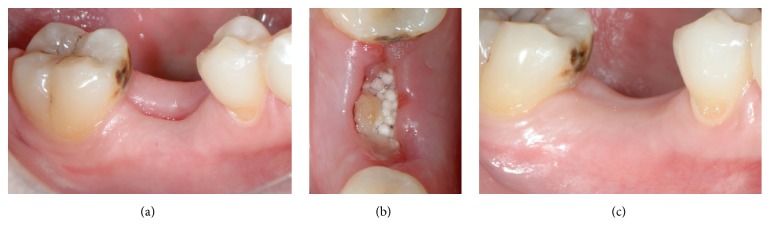
Soft tissue healing. (a, b) Clinical view 1 week post-op. The hemostatic dressing material at the top of the exposed grafted socket is already washed out. However, the grafting material is stable and the soft tissue starts to proliferate over it; (c) clinical view 4 months post-op. The site is covered with newly formed epithelium, and the buccal keratinized soft tissue and the dimensions of the ridge are adequately preserved, while the mucogingival junction is not displaced.

The following procedure was planned for all sites. Tooth extraction was performed under local anaesthesia without flap elevation. Periotomes and forceps were gently used. In order to minimize surgical trauma, multirooted teeth were sectioned with a Lindemann burr (Komet Inc., Lemgo, Germany) under copious irrigation with sterile saline, and each root was independently mobilized and carefully luxated (Figure 2). Attention was given not to damage the surrounding soft and hard tissues, especially in the buccal aspect. All sockets were thoroughly curetted to remove granulation tissue, followed by rinsing with sterile saline. A periodontal probe was then utilized to explore the buccal plate (Figure 3(a)) which had to be in all cases intact (four-wall postextraction sockets). A fully resorbable alloplastic* in situ *hardening bone substitute (*easy-graft™* CLASSIC, Sunstar GUIDOR, Etoy, Switzerland) was used to graft the site according to the manufacturer's instructions (Figure 3(b)). The material consists of *β*-TCP granules which are coated with a thin (10 *μ*m) layer of polylactide (PLGA) and preloaded in a plastic sterile syringe. According to the manufacturer's instructions, prior to injecting the material into the socket the granules are mixed in the syringe with the provided liquid Biolinker*™* (N-methyl-2-pyrrolidone solution). The Biolinker turns the coated granules to a sticky mass which, after application in the socket and upon contact with blood, begins to progressively harden* in situ*. A bone plunger was used to condense and shape the sticky easily handling mouldable graft granules in order to occupy all the volume of the socket up to the level of the surrounding host bone (Figure 3(c)). Attention was given not to overfill the socket as this could result in subsequent sequestration of the exposed coronal granules or displacement of the entire graft mass after mechanical irritation during the first phases of healing. A saline-wet gauze was used to further compact the granules and accelerate the hardening of the graft* in situ* so that after a few minutes the alloplastic bone substitute formed a stable, rock-solid, porous scaffold for the host osseous regeneration. The site was then covered with a haemostatic dressing material (Jason® Collagen Fleece, Botiss) and a cross-mattress tension-free 5/0 suture (Seralon® Serag-Wiessner KG, Naila, Germany) was placed over to achieve soft tissue stability (Figures 3(d)–3(f)). All sites were left uncovered without obtaining primary closure in order to heal by secondary intention. The patients did not wear any prosthesis during the healing period and smoker patients were advised to avoid smoking for at least 1 week post-op. Antibiotic therapy consisting of 1 g amoxicillin every 12 hours for 4 days and mouth rinsing with 0.2% chlorhexidine every 8 hours for 10 days were prescribed. The suture was removed 1 week postoperatively (Figures 4(a) and 4(b)).

**Figure 5:**
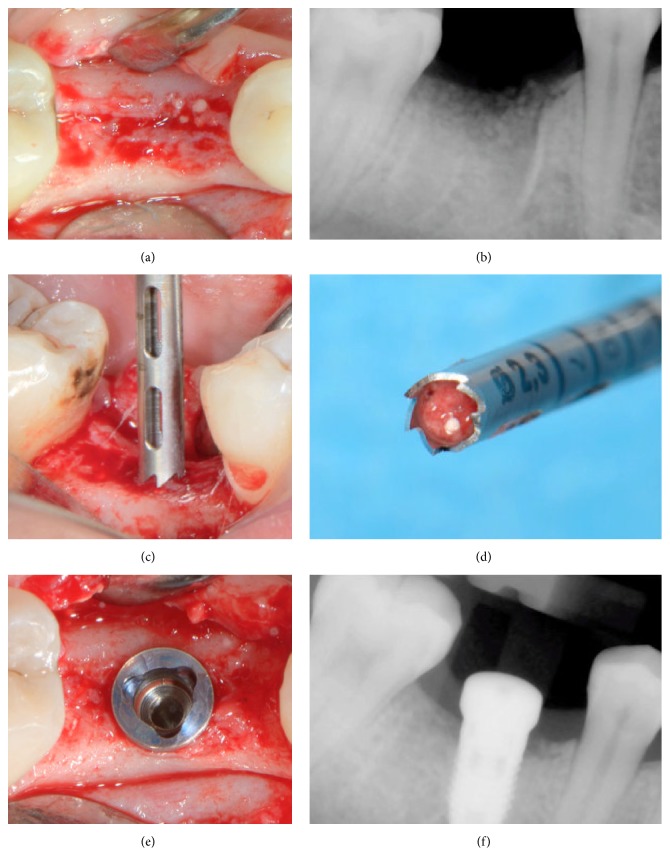
Surgical reentry at 4 months. Intraoperative picture (a) and periapical X-ray (b) showing new bone formation; trephine biopsy (c, d); implant placement at the optimal positioning (e, f).

After 4 months, the sites were reentered for implant placement (Figures 4(c), 5(a), and 5(b)). A site-specific full thickness mucoperiosteal flap was elevated to expose the regenerated hard tissue. A bone core biopsy was taken with a minimum depth of 7 mm from the centre of the site (Figures 5(c) and 5(d)) using a trephine drill with a diameter of 2.3 mm (Komet Inc., Lemgo, Germany). Following the harvesting of the bone sample, the preparation of the bony bed was completed at the same site and an implant was placed (NobelReplace*™* Tapered, Nobel Biocare AB, Göteborg, Sweden) according to the manufacturer's surgical protocol (Figures 5(e) and 5(f)). Immediately after placement, the final seating torque was recorded using the manufacturer's hand ratchet (Nobel Biocare AB, Göteborg, Sweden) and the initial stability was also measured by resonance frequency analysis (Osstell ISQ*™*, Göteborg, Sweden). For each implant, 2 ISQ measurements were recorded, palatally (or lingually) and mesially, according to the guidelines of the company. The mucoperiosteal flap was closed with interrupted resorbable 4-0 sutures (Vicryl®, Ethicon, Johnson & Johnson, Somerville, NJ, USA).

### 2.3. Histological and Histomorphometrical Evaluation

The reference area for the histomorphometrical evaluation was the entire area in the biopsy. Values measured in % of the examined area were taken for biomaterial, old bone, and newly formed bone. The trephine burs with the bone biopsies inside were fixed in four percent formalin for 5–7 days, rinsed in water, and dehydrated in serial steps of ethanol (70%, 80%, 90%, and 100%), remaining for one day in each concentration. Specimens were then infiltrated, embedded, and polymerized in resin (Technovit 9100, Heraeus Kulzer, Wehrheim, Germany) according to the manufacturer's instructions. After polymerization, samples were cut in 500 *μ*m sections using a precision cutting machine Secotom 50 (Struers, Ballerup, Denmark). The sections were mounted on acrylic slides (Maertin, Freiburg, Germany) and ground to a final thickness of approximately 60 *μ*m on a rotating grinding plate (Struers, Ballerup, Denmark). Specimens were subsequently stained with Azur II and Pararosanilin (Merck, Darmstadt, Germany), which allowed for a differentiation between graft granules, preexisting and newly formed bone. Imaging was performed with an Axio Imager M1 microscope equipped with a digital AxioCam HRc (Carl Zeiss, Göttingen, Germany). Histomorphometric analysis was performed by one observer digitally using the analySIS FIVE software (Soft Imaging System, Münster, Germany).

### 2.4. Statistics

Data were expressed as mean ± standard deviation (SD). Pearson correlation coefficient was used to analyse the relation between the quantitative measures and statistical significance was set at* p* < 0.05. All analyses were carried out using the statistical package SPSS ver. 17.00 (Statistical Package for the Social Sciences, SPSS Inc., Chicago, IL, USA).

## 3. Results

The final results are shown in Tables 1 and 2. Ten patients (8 women and 2 men) with a mean age of 45.7 years (range: 23 to 76 years) participated in this prospective case series. Of the 10 sites, 4 were in the molar region of the mandible, 3 were in the molar region of the maxilla, 2 were in the premolar region of the maxilla, and 1 was in the anterior region of the mandible. Three of the 10 patients were smokers.

**Figure 6:**
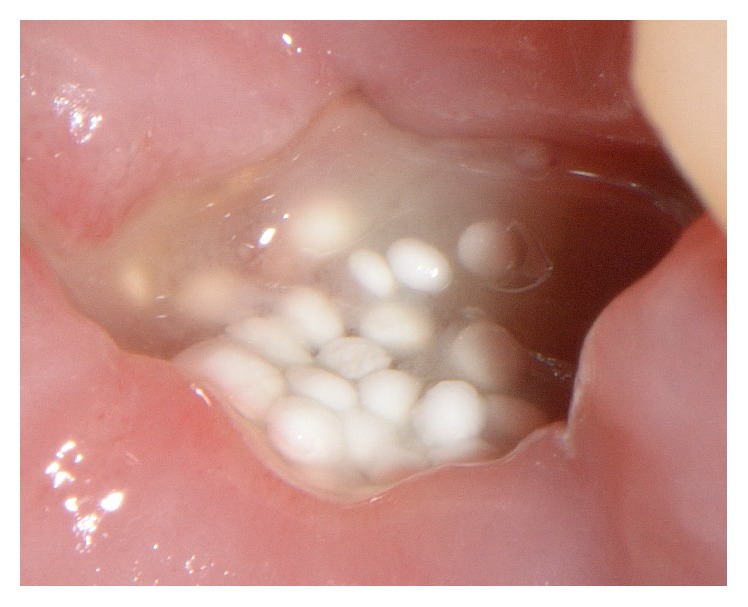
One week of secondary intention healing after socket grafting.

**Figure 7:**
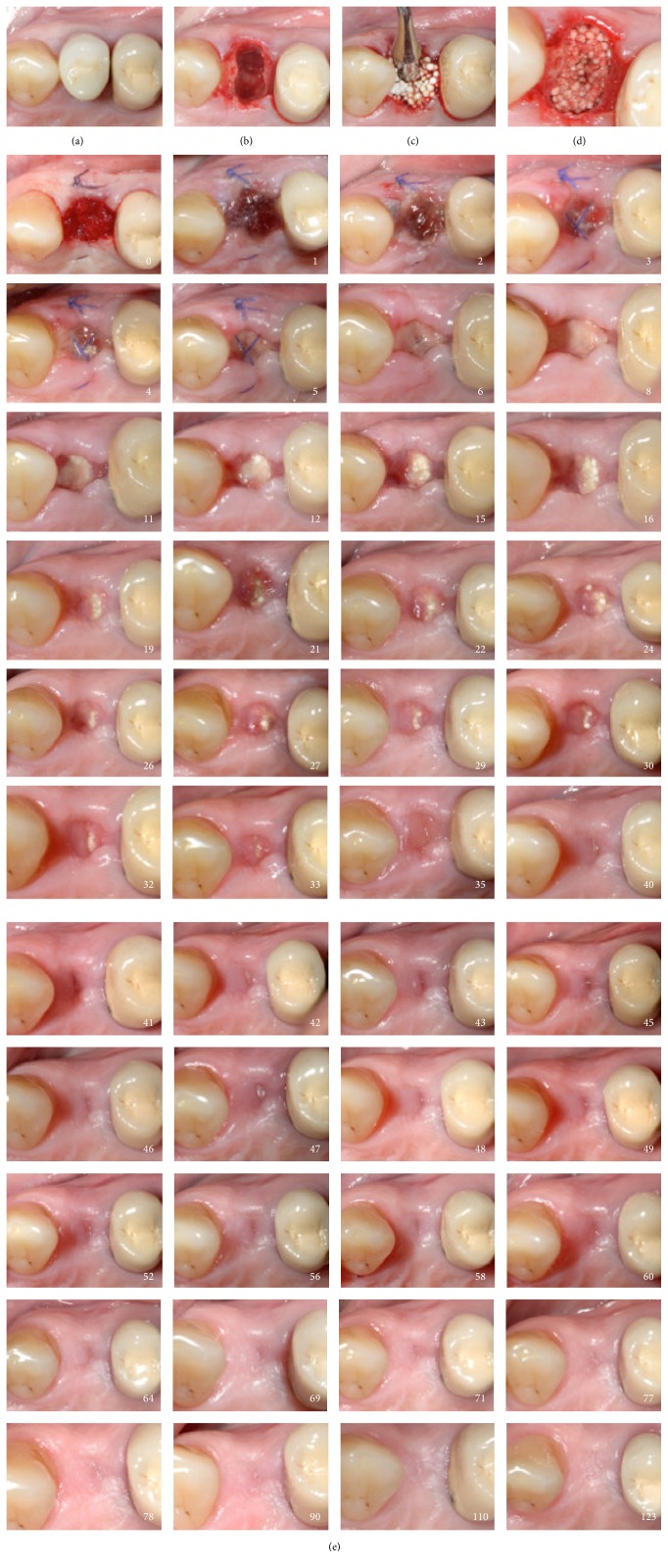
Secondary intention healing cascade. Tooth number 24 (a) was extracted (b) and grafted (c, d) with no primary closure (e) according to protocol. White numbers indicate the postoperative day.

**Figure 8:**
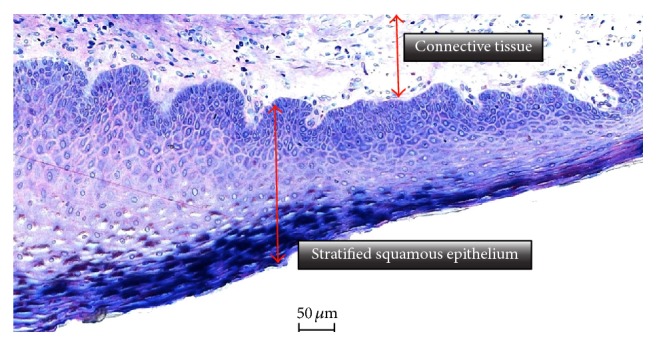
Histological picture showing that the newly formed soft tissue at the top of the ridge is composed of connective tissue covered by regular stratified squamous keratinized epithelium (Azur II and Pararosanilin; original magnification ×50).

In all cases, the postoperative healing was uneventful. Clinically, the same soft tissue healing pattern was observed: The hemostatic dressing material at the top of the exposed grafted socket was washed out in the first 2 days postoperatively. However, no loss or sequestration of bone graft granules was observed during the healing period although the sites were then left uncovered. Due to the specific* in situ* hardening biomechanical properties of the grafting material, the stable granules provided a solid immobile scaffold over which newly formed soft tissue migrated from the margins of the sockets, achieving secondary intention soft tissue healing. In particular, in the first stage graft particles were embedded in fibrin matrix that was slowly replaced by a layer of connective tissue, which started to cover the exposed stable graft granules from the wound edges. Subsequently, epithelial cells from the periphery proliferated over this connective tissue layer (Figures 6 and 7) and after 4 months all areas were covered with newly formed keratinized epithelium. At this time point, clinical examination showed that the volume and architecture of the ridges were adequately preserved. In one patient, a small biopsy of the newly formed soft tissue at the top of the ridge was harvested prior to reentry. Histological examination showed that the socket was covered after 4 months by keratinized epithelium, identical to the surrounding host soft tissues (Figure 8).

At reentry on the day of implant placement after 4 months the grafted areas were filled with newly formed bone. At sites, residual particles were visible, embedded, and in continuity with the regenerated hard tissue. All implants were placed at the precise 3D position showing excellent initial stability in the majority of the cases. At implant placement, the mean final seating torque value was 43.6 ± 8.3 Ncm, and the mean ISQ measurements were 69 ± 11.1, showing excellent initial stability for the majority of the cases. There was a significantly strong correlation between final seating torque values and ISQ values (Table 2).

**Figure 9:**
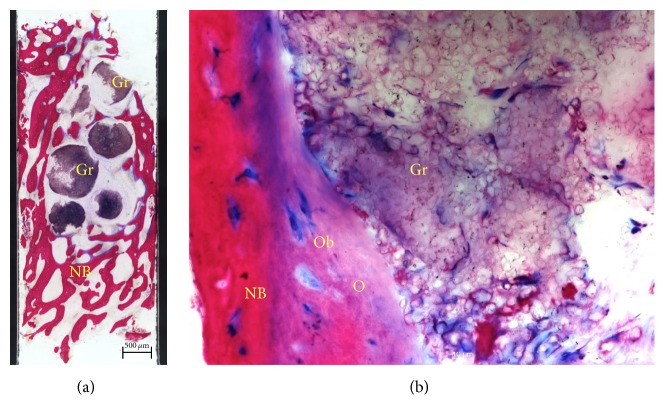
Histological examination. Saw cut (a) of the core biopsy showing formation of new bone (NB). Graft particles (Gr) are embedded in connective tissue and bone trabeculae (Azur II and Pararosanilin, original magnification ×50); (b) osteoblasts (Ob) forming osteoid (O) and adding new bone (NB) to the surface of partially disintegrated graft particles (Gr) (Azur II and Pararosanilin, original magnification ×200).

Histologically, all analysed biopsies contained newly formed bone, residual graft material, and well vascularized uninflamed connective tissue (Figure 9). No necrosis or foreign body reactions were detected. The graft granules were surrounded by or in contact with active osteoblasts forming osteoid and new woven bone, demonstrating persistent osteogenesis. At this time point, the graft particles were partially disintegrated showing cellular infiltrates of lymphocytes and basophil, while being integrated in newly formed bone. Resorbing granules of the grafting material could also be identified embedded in connective tissue with no histological signs of inflammation. In many areas, active bone remodeling was evident with mature lamellar bone replacing the woven bone.

Histomorphometric analysis revealed that after 4 months of healing new bone represented 24.4 ± 7.9% of tissue and residual grafting material represented 12.9 ± 7.7% of tissue, while 60.5 ± 7.4% was occupied by connective tissue/bone marrow.

**Figure 10:**
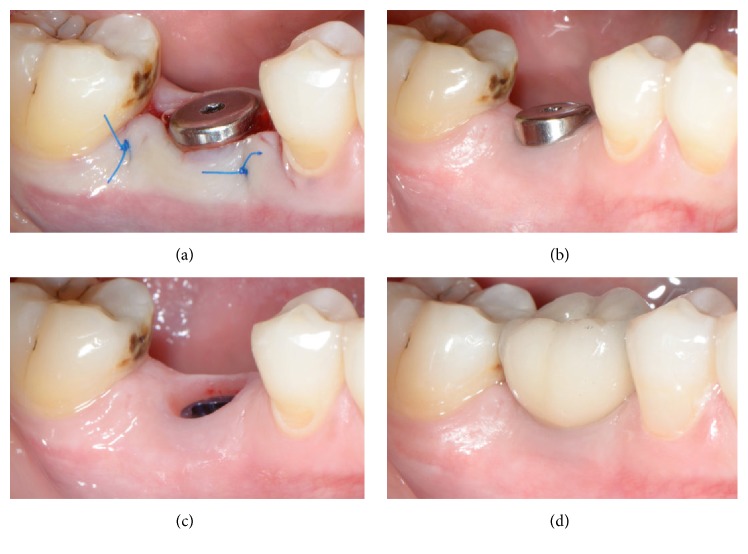
Restorative phase. The newly formed keratinized soft tissue at the top of the site could be pushed buccally (a) in order to enhance the soft tissue contour (b, c) of the final restoration (d).

In all cases, 2 months after implant placement a vertical crestal incision was made and a healing abutment was placed (Figures 10(a) and 10(b)). After allowing 2 weeks for the maturation of the soft tissues (Figure 10(c)), the final restoration was fabricated with successful functional and aesthetic results (Figure 10(d)).

## 4. Discussion

This case series reports on the application of an* in situ* hardening resorbable alloplastic grafting material in 10 cases of alveolar ridge preservation and subsequent implant placement.

A routine but minimally invasive surgical protocol was followed in all cases. Extractions and grafting were performed without raising a flap. After grafting, the augmented sites were not covered with a barrier membrane or an advanced buccal flap. Elevating the periosteum from the buccal bone to create a mucoperiosteal flap will compromise the blood supply of the exposed bone surface, leading to osteoclastic activity and increased bone resorption [26]. Moreover, this approach was selected in order to minimize patient morbidity, surgical time, and cost, but mostly in an attempt not to displace the mucogingival junction and to allow for the spontaneous formation of new keratinized soft tissue over the postextraction grafted site. According to the clinical findings of this report, it seems that secondary intention soft tissue healing of grafted postextraction sites can be achieved when filling the sockets with self-stabilizing solid bone substitutes. The absence of unwanted soft tissue ingrowth into the grafted site was verified in all cases clinically at reentry and also histologically. Our findings are in accordance with those reported by Jurišić et al. [22] who filled postextraction sockets with* in situ* hardening biphasic calcium phosphate granules coated with PLGA without achieving primary closure or covering the site with a barrier membrane. The authors found that all treated sites healed uneventfully with spontaneous healing of the socket opening, with no local complications and infections or patient discomfort during an observation period of 4 months. From all the above findings, it can be postulated that the addition of PLGA to the graft results in a stable, solid alloplastic bone substitute, deterring the loss of exposed granules and at the same time serving as a barrier membrane for blockage of soft tissue ingrowth. In contrast, a flap and/or a barrier membrane may be necessary to provide the necessary stability and protection to loose particulate grafts [27] without self-hardening characteristics. The argument of implementing flapless techniques when possible is also supported by the conclusions of a systematic review by Wang and Lang [12]. The authors, analysing the existing evidence, reported that achieving primary closure did not present beneficial effects on preserving the ridge width; on the other hand, with primary flap closure, patients experienced more discomfort and the mucogingival junction was significantly more coronally displaced. In the present report, all sites were spontaneously fully covered with newly formed keratinized soft tissue while the buccal keratinized soft tissues were preserved after 4 months. As the presence of an adequate zone of keratinized gingiva is an important parameter in achieving esthetic implant restorations, preventing future mucosal recessions, and improving the overall long-term implant stability [28, 29], the use of flapless techniques by taking advantage of the biomechanical properties of* in situ* hardening alloplastic grafts seems to be a benefit for the clinicians and patients when applicable.

In all our cases, the dimensions of the ridge were adequately preserved after 4 months. However, in this case series no volumetric measurements were made in order to evaluate the horizontal and vertical dimensional changes of the alveolar ridge. At reentry all sites were filled with regenerated bone, allowing for the precise placement of an implant at the optimal positioning. The histomorphometrical analysis of the harvested samples revealed pronounced new bone formation with 24.4 ± 7.9% of tissue occupied by newly formed vital bone. In 2013 Schmidlin et al. [21] evaluating* in situ *hardening *β*-TCP granules coated with PLGA in surgically created calvaria defects in rabbits reported 18.2 ± 5.8% of new bone after 16 weeks of healing.

Histology showed that only small amounts of residual grafting material were present after 4 months in all cases. According to the histomorphometrical analysis, only 12.9 ± 7.8% of the newly formed hard tissue was occupied by still nondissolved material, which is significantly lower than the 40% limit set for obtaining successful and predictable implant placement [30, 31]. The grafting material used in the present cases consists of *β*-TCP and PLGA, which are fully dissolved after 9–12 months [16, 18–20, 32–36]. For this reason, it can be postulated that the remaining particles, observed clinically and histologically at 4 months, will be eventually completely replaced by vital bone, restoring and reestablishing the original biomechanical properties of the regenerated bone. Residual particles remaining at the implant site from a poorly resorbable material may potentially change the natural biomechanical properties of the bone into which it has been implanted [37]. In particular, the presence of residual nonresorbable or slowly resorbable graft particles might interfere with normal bone healing and remodeling and it is possible to affect the degree of bone-to-implant contact, or the initial stability of the implant placed in the augmented site. A recent systematic review by Chan et al. [8] reported conflicting results with the use of xenografts, with changes in the percentage of vital bone ranging from −22% (decrease) to 9.8% (increase), while considerable residual HA and xenograft particles (15% to 36%) remained at a mean of 5.6 months after socket grafting procedures. Although it remains unknown whether these changes in bone quality will affect implant success and peri-implant tissue long-term stability, it is without doubt that bone quality is of paramount importance in successful implant therapy [38]. Mordenfeld et al. [13] analysing biopsies harvested from sinuses augmented with anorganic bovine bone and autogenous bone showed the presence of nonresorbed intact xenograft particles with no significant changes in particle size after 11 years. Jensen et al. [39] compared the bone formation and graft resorption at surgically created mandibular standardized osseous defects in minipigs grafted with anorganic bovine bone, *β*-TCP, or autogenous bone. After 8 weeks of healing they found that more bone was regenerated in defects filled with *β*-TCP or autogenous bone compared to those filled with xenograft. In parallel autograft particles and *β*-TCP were almost completely resorbed whereas anorganic bovine bone showed no evidence of resorption.

Moreover, the improved stability throughout the graft material seems to further improve the quality of the bone to be regenerated due to reduced micromotion of the graft particles. It is known that micromovements between bone and any implanted grafted material prevent bone formation and may lead to mesenchymal cell differentiation to fibroblasts instead of osteoblasts, resulting in the development of fibrous tissue [40, 41]. The impact of grafted site stability on bone healing was discussed by Troedhan et al. [42] who evaluated the initial stability of implants inserted in sinus-lift sites augmented with* in situ* hardening alloplastic bone substitutes, or loose particulate biomaterials. The authors reported significantly better results in sites regenerated with* in situ* hardening alloplasts arguing that the self-hardening properties of these bone grafting materials allowed improved vascularisation and mineralization of the subantral scaffold by full immobilization of the augmented site towards pressure changes of the sinus at normal breathing.

The results of this case series also revealed a significant linear correlation between final seating torque and ISQ, indicating adequate primary stability of the implants placed at the grafted sockets 4 months post-op. This is in agreement with the findings of previous studies reporting that higher insertion torque values resulted in greater ISQ measurements [23, 43–45]. It is important that primary stability is not only affected by the geometry of the implant placed (i.e., length, diameter, and type) and the placement technique used (relation between drill size and implant size, tapping) but also positively related to the quality, density, and quantity of the local bone [23]. Considering that in the present case series the same implant type and technique were utilized in all cases, the reported high primary stability values might suggest good quality of the regenerated bone at 4 months post-op.

## 5. Conclusions

The results of this clinical case series support the use of an* in situ* hardening alloplastic resorbable grafting material for bone regeneration in postextraction sites. Grafting of sockets without primary wound closure can be an effective minimally invasive method of preserving the contour and architecture of the alveolar ridge. The hardening characteristics of the grafting material used seem to be of great importance for the stability of the healing site and the success of the above technique. Additional prospective studies using control groups, larger patient populations, and other time frames are needed in order to confirm and supplement the present findings.

## Figures and Tables

**Table 1:** Patients' characteristics, volume densities of new bone, residual graft and connective tissue/bone marrow in biopsy specimens, and ISQ and final seating torque measurements.

Cases	Age	Sex	Smoker	Site	New bone volume (%)	Graft volume (%)	Connective tissue/bone marrow (%)	ISQ		Final seating torque (Ncm)
								Buccal-lingual	Mesial-distal	
1	23	F	No	15	27.4	15.6	57	73	73	50
2	49	F	Yes	36	18.7	6.7	72.7	75	75	50
3	50	F	No	46	15.8	20.2	60.2	79	79	50
4	29	F	Yes	46	40.5	7.2	52.3	79	79	50
5	57	M	No	16	31.7	2.7	58.7	70	75	45
6	56	F	No	37	20.5	27.4	50.6	70	75	45
7	48	M	No	17	29	4.2	66.8	49	55	32
8	46	F	Yes	24	22	13.1	62.2	71	73	50
9	76	F	No	17	23.5	17	54.4	56	58	32
10	23	F	No	31	14.5	15.3	70	49	48	32
*Mean*	*45.7*				*24.36*	*12.94*	*60.49*	*67.1*	*69*	*43.6*
*SD*	*16.65*				*7.95*	*7.78*	*7.45*	*11.50*	*11.05*	*8.25*

**Table 2:** Statistically significant correlation between ISQ measurements and final seating torque.

		Final seating torque (Ncm)
ISQ Buccal-lingual measurement	Pearson correlation (*r*)	**0.963**
	*p* value	<0.0005
	*N*	10

ISQ Mesial-distal measurement	Pearson correlation (*r*)	**0.937**
	*p* value	<0.0005
	*N*	10
